# Nitrogen concentration shapes the size structure and the functional diversity of phytoplankton communities in the southern Indian Ocean

**DOI:** 10.1093/ismeco/ycaf195

**Published:** 2025-11-20

**Authors:** Hugo Berthelot, Joanna Zukowska, Nicolas Henry, Cyril Noël, Melilotus Thyssen, Karine Leblanc, Hélène Planquette, Jean-François Maguer, Rainer Pepperkok, Colomban de Vargas, Nicolas Cassar

**Affiliations:** CNRS, Université de Brest, IRD, Ifremer, LEMAR, F-29280 Plouzané, France; Cell Biology and Biophysics Unit, European Molecular Biology Laboratory, 69117 Heidelberg, Germany; Now at Ifremer, DYNECO, Pelagos Laboratory, F-29280 Plouzané, France; Cell Biology and Biophysics Unit, European Molecular Biology Laboratory, 69117 Heidelberg, Germany; CNRS, Sorbonne Université, FR2424, ABiMS, Station Biologique de Roscoff, Roscoff 29680, France; Research Federation for the Study of Global Ocean Systems Ecology and Evolution, FR2022/GOSEE, Paris 75016, France; Ifremer, IRSI, SeBiMER Service de Bioinformatique de l'Ifremer, F-29280 Plouzané, France; Aix Marseille University, Université de Toulon, CNRS, IRD, MIO UM 110, 13288 Marseille, France; Aix Marseille University, Université de Toulon, CNRS, IRD, MIO UM 110, 13288 Marseille, France; CNRS, Université de Brest, IRD, Ifremer, LEMAR, F-29280 Plouzané, France; CNRS, Université de Brest, IRD, Ifremer, LEMAR, F-29280 Plouzané, France; Cell Biology and Biophysics Unit, European Molecular Biology Laboratory, 69117 Heidelberg, Germany; CNRS, Sorbonne Université, FR2424, ABiMS, Station Biologique de Roscoff, Roscoff 29680, France; Research Federation for the Study of Global Ocean Systems Ecology and Evolution, FR2022/GOSEE, Paris 75016, France; CNRS, Université de Brest, IRD, Ifremer, LEMAR, F-29280 Plouzané, France; Division of Earth and Climate Sciences, Nicholas School of the Environment, Duke University, 27708 Durham, NC, United States

**Keywords:** phytoplankton, nitrogen, cell size structure, mixotrophy, diazotrophy, southern Indian Ocean

## Abstract

Phytoplankton are fundamental to marine ecosystems, biogeochemical cycling and climate regulation. Their community structure and productivity are shaped by biotic and abiotic factors, notably temperature and macronutrient concentrations. Climate change is altering ocean vertical stratification and nutrient dynamics, with complex and often poorly understood impacts on phytoplankton communities and global primary production. To contribute characterizing these relationships, we analysed planktonic community composition using 18S rRNA amplicon sequencing and imaging flow cytometry in the southern Indian Ocean across a strong environmental gradient from warm, stratified, N-depleted (but relatively P-repleted) waters in the north to cold, mixed, macronutrient-replete waters in the south. Phytoplankton composition and local diversity correlated primarily with temperature and macronutrient concentrations, but smaller cells (<3 μm) were less affected than larger ones (>3 μm). To disentangle the relative influence of temperature and macronutrients, we applied a model of dissolved macronutrient diffusion, suggesting that nutrient limitation, primarily nitrogen, likely constrains the growth of osmotrophic phytoplankton with cell sizes exceeding 2-15 μm in the nutrient-depleted region. We show that smaller cells, with higher surface area-to-volume ratios, are likely to evade this limitation, explaining their lower sensitivity to nitrogen concentrations, both in their taxonomic composition and diversity. Imaging flow cytometry confirmed that larger cells persisting in nitrogen-depleted waters predominantly employ alternative nitrogen acquisition strategies such as diazotrophy or mixotrophy, fostering functional local diversity. Notably, three Prymnesiophyceae taxa exhibited partial limitation by nitrogen diffusion, raising questions about their potential for mixotrophy or diazotrophy, akin to *Braarudosphaera bigelowii*. Other environmental factors, such as trace metal concentrations, showed weaker correlations with community structure metrics. Overall, our results are consistent with N concentration gradients and N:P imbalances driving a great share of planktonic diversity by constraining large-cell nutrient acquisition strategies and fostering functional diversification in oligotrophic regions of the Ocean.

## Introduction

The ocean harbors a vast diversity of phytoplankton, which are microscopic photosynthetic organisms playing a crucial role in marine ecosystems and climate. These microorganisms provide critical ecosystem services by contributing to the biogeochemical cycling of climate-relevant elements, such as carbon, nitrogen, and phosphorus, and by serving as the foundation of marine trophic food webs [1, 2]. Their productivity and dynamics significantly influence oceanic carbon sequestration, global food security, and biodiversity [3, 4]. However, ongoing global climate change has profoundly impacted ocean circulation, temperature, pH, light conditions, and nutrient concentrations, with poorly characterized effects on phytoplankton communities and marine productivity [5–7]. Characterizing the factors controlling phytoplankton community structure and their ecological functions in the face of these changes has become an urgent scientific challenge, as it is crucial for projecting future changes in ocean biomes and climate.

Among the numerous factors influencing phytoplankton communities, temperature and macronutrient concentrations (e.g. nitrogen and phosphorus) are the most extensively studied [8–11]. Both factors directly and indirectly shape phytoplankton community composition and diversity. Temperature, in particular, exerts a multifaceted influence through physiological and ecological mechanisms. For example, species-specific thermal traits and temperature-dependent biotic interactions, such as grazing and viral infections, are known to determine the biogeography of phytoplankton species [12]. In situ and culture experiments demonstrate that the distribution of cyanobacteria clades, such as *Prochlorococcus* (the smallest yet most abundant phytoplankton), is partially controlled by temperature [13, 14]. Similar temperature-driven niche partitioning is observed in clades of other widely distributed phytoplankton species, such as *Synechococcus* and *Ostreococcus* [15, 16]. These findings are further corroborated by global modeling efforts, which confirm temperature’s critical role in shaping plankton biogeography [17].

Temperature also influences phytoplankton diversity. A pronounced latitudinal diversity gradient is observed in the ocean, with alpha diversity generally declining toward the poles. This gradient is strongly associated with temperature [7, 18, 19], though the underlying mechanisms remain debated. Two prominent hypotheses have been proposed: the “physiological tolerance hypothesis” suggests that thermal sensitivity spectra are skewed toward higher temperatures, limiting species survival in colder regions [9, 20, 21]. Meanwhile, the “kinetic energy hypothesis” posits that higher temperatures increase metabolic rates, ultimately leading to faster speciation rates [22, 23]. These mechanisms may interact with other factors, such as light availability and nutrient gradients, to drive observed patterns of diversity [24].

Nutrient concentration is another critical determinant of phytoplankton community structure. Nutrient concentrations and chemical forms strongly influence species composition, abundance, and productivity. Margalef’s mandala, a pioneering conceptual framework, proposed that phytoplankton community strategies are defined along two axes: turbulence and macronutrient concentration [25]. According to this model, diatom-dominated communities prevail in high-macronutrient, turbulent environments, whereas dinoflagellates dominate in low-macronutrient, stratified conditions typical of late growing seasons [25]. This framework has been refined to include additional parameters, such as nitrogen forms (reduced versus oxidized), contrasting nitrogen-to-phosphorus ratios, temperature, and functional traits like cell size, motility, mixotrophy, and toxin production [26]. Gilbert [27] expanded the mandala by incorporating these parameters while maintaining nutrients as a primary driver of community composition. Short-term nutrient enrichment experiments in nutrient-depleted ocean regions have confirmed the importance of nutrient concentration and chemical form in shaping plankton communities. For instance, nutrient addition experiments in the North Pacific Subtropical Gyre have shown dramatic increases in plankton productivity and shifts in community composition following nitrogen or phosphorus enrichments [28, 29]. On a larger scale, ocean-wide surveys have revealed that the biogeography of *Prochlorococcus* clades aligns with their genotypic capacities to cope with nutrient stress, highlighting the interplay between nutrient concentration and phytoplankton diversity [30]. In addition, nutrient concentration can also affect plankton size distribution: small cells, with high surface area to volume ratio (SA/V) are theoretically advantaged in dissolved resource acquisition. Nutrient limited environments, this would leads to the exclusion of large osmotrophic cells by the more competitive small osmotrophic cells, a process which has long been advanced to explain their dominance in subtropical gyres [31–33]. Yet, the validity of this resource-based competitive exclusion has recently been debated [34, 35] and alternative models based on “competition-neutral resource landscape” has been proposed where the phytoplankton size distribution is rather driven by environmental stability and phytoplankton-herbivore interactions [36].

Despite advances in understanding temperature and nutrient controls on phytoplankton community structure, disentangling their relative influences at ocean basin scales remains challenging. This difficulty arises from their strong negative correlation: in the sunlit upper ocean, the highest temperatures are typically associated with stratified, oligotrophic conditions, while lower temperatures are linked to nutrient-rich, mixed layers. As global climate change intensifies, ocean stratification is projected to increase, reducing nutrient supply to surface waters and potentially leading to a general decline in primary production in low latitude [37–40]. Numerical models predict shifts in phytoplankton community composition and biogeography driven by these changes, with cascading effects on marine ecosystems and carbon cycling [4, 41].

To address the relative influence of temperature and nutrient on plankton dynamics, we characterized the planktonic community composition using amplicon sequencing of the 18S rRNA gene and automated imaging flow cytometry collected during an inter-basin cruise in the southern sector of the Indian Ocean. The results indicate that macronutrients exert a critical control on structure, composition and functional diversity of phytoplanktonic communities which are cell-size dependent. These results underscore the necessity of integrating multiple approaches to unravel the complex interactions between temperature, nutrients, and other environmental factors.

## Material and methods

**Figure 1:**
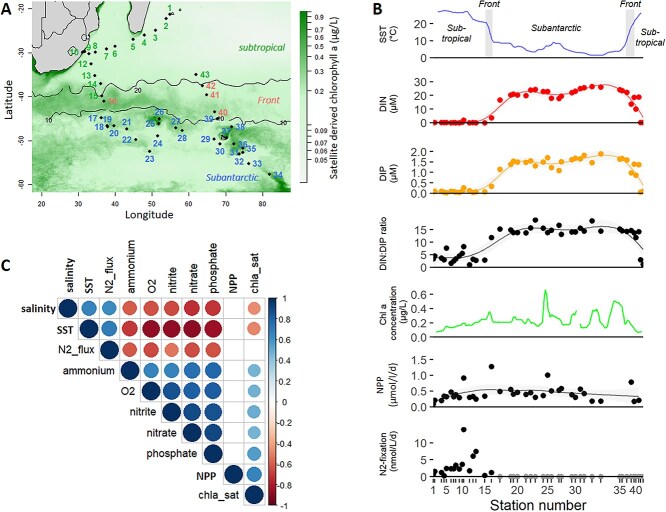
(A) Map of the 43 stations sampled overlaid on average satellite chlorophyll a (Chl a) concentrations (μg.L^−^1) during the cruise (AQUA/MODIS, composite image of January/February 2021). Black lines depict the isothermal sea surface temperatures of 10°C and 20°C defining the subtropical, front and subantarctic regions in this study. (B) Main environmental parameters measured along the cruise track. Sea surface temperature (SST) and Chl a were measured continuously by the underway system equipped with a temperature probe. Samples for dissolved inorganic N (DIN, defined as the sum of nitrate, nitrite and ammonium), dissolved inorganic P (DIP), net primary production (NPP) and N_2_-fixation were acquired discretely (dots). For N_2_-fixation, gray dots denote rates below detection levels. For DIN, DIP and DIP:DIP ratio and NPP, the lines are the modeled values from a general additive model fitted to discrete samples. (C) Heatmap of the correlations between environmental variables (spearman correlation, only significant correlation at the threshold of *p* < 0.005 are shown).

This study was carried out from 13 January to 4 March 2021 (austral summer) aboard the R/V Marion Dufresne II, as part of the SWINGS cruise (GEOTRACES GS02) (doi: 10.17600/18001925) (Fig. 1). Surface seawater was collected at eight stations using Niskin bottles mounted on a conductivity-temperature-depth (CTD) rosette and at 43 additional stations using an underway sampling system (Fig. 1). Underway samples were collected from the ship’s hull intake through the moonpool with a polytetrafluoroethylene (PTFE) diaphragm pump and tubing to minimize trace metal contamination. Sea surface temperature and salinity data were obtained from the CTD sensors, while chlorophyll a concentrations (hereafter Chl a) were derived from Aqua MODIS satellite data (from 13 January to 4 March 2021, L3M 4 km product). Samples for macronutrients and trace metals were collected using a trace-metal-clean, polyurethane-coated aluminum rosette equipped with 24 externally closing 12-liter Teflon-lined GO-FLO bottles (General Oceanics) mounted on a CTD frame and attached to a Kevlar® line, in accordance with GEOTRACES protocols. Seawater samples for nitrate, nitrite, ammonium, and phosphate (later referred to as Dissolved Inorganic Phosphorus, DIP) were collected and filtered through 0.2 μm polycarbonate filters and stored at −20°C until analysis using a SEAL analytical segmented flow analyser, with detection limits of 0.03 μmol L^−1^ for nitrate, 0.01 μmol L^−1^ for nitrite, and 0.01 μmol L^−1^ for DIP according to [42]. Seawater samples for silicic acid were pre-filtered through 0.2 μm polycarbonate filters, preserved with HgCl_2_ (4 mg L^−1^), and stored in the dark at room temperature until analysis using a Skalar segmented flow autoanalyser. The detection limits were 0.07 μmol L^−1^. Samples for trace metals were analysed following Tonnard et al. [43]. Net primary production (NPP) and N_2_ fixation rates were measured using dual labelling isotope ^13^C/^15^N assays (see supplementary material for details). Size fractionated phytoplankton community structure was characterized by amplicon sequencing of 18S rRNA genes (see supplementary material for details). Raw data were deposited on the European Nucleotide Archive (ENA, https://www.ebi.ac.uk/ena) with the accession number PRJEB89894. Phytoplankton cells were imaged using an automated imaging flow cytometer (CytoSense CytoBuoy) [44] installed on an independent underway sampling system, connected to the vessel thermosalinograph pump, acquiring samples every 2 h [45]. Objects were segmented and morphological features were extracted using “EBImage” R package (see supplementary material for details). Images were deposited on Ecotaxa (https://ecotaxa.obs-vlfr.fr/gui/prj/13402).

As phytoplankton cellular size increases, their SA/V tends to decrease (for a sphere, SA/V = 3/radius), which eventually limits osmotrophic dissolved nutrient acquisition, especially at low nutrient concentrations. Nutrient diffusion models are based on the principle that the growth of purely osmotrophic organisms cannot exceed their capability to encounter dissolved nutrients. Thus, the smaller the cell, the higher the SA/V, and the higher the potential osmotrophic growth rate. Similarly, the lower the nutrient concentrations, the lower the potential for osmotrophic growth rates. It results in a maximum osmotrophic growth rate depending on both cell size and nutrient concentrations. The maximal osmotrophic nutrient fluxes constrained by diffusion-limited nutrient supply to single cells were calculated from the analytical solution of diffusion to a sphere according to the mass transfer theory described in Sherwood et al. [46] as follows:


(1) \begin{equation*} {\rho}_{Nmax}=4\pi{Dr}_0\left({C}_{\infty }-{C}_0\right) \end{equation*}

where ρ_Nmax_, the maximal dissolved nutrient uptake rate (nmol s^−1^) of a cell with the equivalent spherical radius r_0_ (cm), D, the molecular diffusion coefficient (cm^2^ s^−1^) of the considered compound in water, C_0_, the dissolved nutrient concentration at the cell surface (assumed to be zero) and C_∞_, the measured concentration in the ambient water (nmol L^−1^). C_∞_ was calculated for each imaged cell with nitrate, nitrite and ammonium concentrations inferred from the nearest stations based on geographical linear interpolation. D was calculated using the “coeffcoeff” function of the “marelac” R package using salinity and temperature measured by the underway CTD probe at the time of cell observation and a pressure of 1 bar [47].

For each image cell, spheroid-based flux were corrected to take into account the elongated nature of the cells (in particular *Trichodesmium* or diatom chains) assuming a prolate spheroid deviating from spheres as described by Jumars et al. [48]:


(2) \begin{equation*} \rho N,\mathit{\max}, corr=\frac{\sqrt{E^2-1}}{\mathit{\ln}\left(E+\sqrt{E^2-1}\right)}\ {\rho}_{Nmax} \end{equation*}

with E, the aspect ratio, defined as length along the axis of symmetry (major axis) divided by diameter orthogonal to that axis (minor axis). The sum of ρ_N,max,corr_ of nitrate, nitrite and ammonium was converted to N-based maximum osmotrophic growth rate (${\rho}_{N_{based, tot}}$, d^−1^) assuming that ammonium can be absorbed independently during day and night and nitrate and nitrite during day only (12-h) and converted to N based maximum osmotrophic growth rates as follows:


(3) \begin{equation*} {GR}_{N\ based}=\frac{\rho_{NH4, NO3,\mathit{\max}}}{N_{cell}} \end{equation*}

With N_cell_, the cellular N content (nmol cell^−1^) calculated according to Verity et al. [49] as follows:


(4) \begin{equation*} {N}_{cell}=\frac{10^{\left(-1.084+0.837.\log BV\right)\Big)}}{M_N.1000} \end{equation*}

with BV, the cellular biovolume (μm^3^ cell^−1^) and ${M}_N$, the N molar mass. P-based maximum osmotrophic growth rates were calculated similarly using DIP concentrations and with P_cell_ = N_cell_ / 16.

## Results

### Biogeochemical setting

We sampled 43 stations in the surface waters of the southern Indian Ocean characterized by a strong latitudinal gradient in temperature and macronutrient concentrations. The northernmost stations displayed temperatures above 20°C (later called subtropical region) and low macronutrient concentrations (sum of nitrate, nitrite and ammonium = 0.25 μM on average, hereafter called dissolved inorganic N, DIN), but an excess in DIP compared to DIN (DIN:DIP = 3.7 on average, ranging 0.9–8.3) with respect to the canonical Redfield ratio (N:P = 16). In contrast, southern stations were characterized by temperatures lower than 10°C (later called subantarctic region), higher macronutrient concentration (DIN > 14 μM) and a DIN:DIP ratio closer to 16 (DIN:DIP = 14.5 on average, ranging 11.5–18.7). Four stations fell in a transition zone (temperature between 10 and 20°C, later called front region) corresponding to the subtropical front with intermediate temperature and macronutrient concentrations. Despite low DIN concentrations in the subtropical region NPP rates were only half (241 ± 189 nmol C L^−1^ d^−1^) those in the subantarctic region (549 ± 478 nmol C L^−1^ d^−1^). Micronutrients were measured at a subset of stations (13 out of 43) and showed relatively weak correlation with the other biogeochemical variables except for Ni and Mn (Fig. S1). NPP did not correlate significantly with any of the environmental parameters measured (except with Chl a, Spearman correlation, *r* = 0.65, *P* = 1.6x10^−5^).

### Phytoplankton community structure

**Figure 2:**
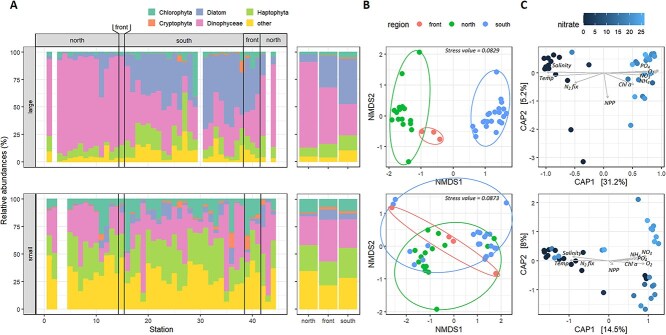
Phytoplankton communities analyzed by amplicon sequencing of the V4 region of the 18S gene in the large (top) and small size fractions (bottom). (A) Relative abundances of the different groups of phytoplankton analyzed by amplicon sequencing of the V4 region of the 18S gene in the large (top) and small size fractions (bottom). The diatom functional group includes the Bacillariophyceae, Coscinodiscophyceae, Diatomeae_X and Mediophyceae. (B) Non-metric multidimensional scaling from bray–Curtis dissimilarity matrix of the phytoplankton communities. (C) Constrained analysis of principal coordinates (CAP) based on the following environmental parameters: Temperature, salinity, nitrate (NO_3_^−^), phosphate (PO_4_^2−^), ammonium (NH_4_^+^), net primary production (NPP), N_2_ fixation rate, chlorophyll a (Chl a) and dissolved O_2_ concentration (O_2_). Parameters not measured systematically at all stations were excluded from the analysis to ensure maximal statistical coverage.

At each of the 43 stations we measured community composition by amplicon sequencing of the V4 region of the 18S rRNA gene. Analysis at the division level shows contrasting patterns between small and large size fractions (Fig. 2A). While the proportion of each photosynthetic phylum doesn’t sensibly change between regions in the small size fraction, a contrasting picture arise in the large size fraction: in the cold and macronutrient replete subantarctic region, Diatoms (functional group including Bacillariophyceae, Coscinodiscophyceae, Diatomeae_X, Mediophyceae) accounts for almost half of the community. In the macronutrient-depleted and warmer region, Diatoms account for less than 10% of the community (except at the coastal stations 10 and 11 near Durban, South Africa) which is largely dominated by Dinophyceae, representing 75% of the community on average. NMDS analysis based on Bray–Curtis dissimilarity of ASVs shows similar patterns with overlapping communities between regions in the small size fraction and totally separated communities between regions in the large size fraction (Fig. 2B). Permanova analysis confirmed a stronger dissimilarity between subtropical and subantarctic regions for large size fractions (*r* = 0.335, *P* = 0.001, pseudo-F = 9.59, degree of freedom = 40 number of permutations = 999) as compared to small size fractions (*r* = 0.141, *P* = 0.001, pseudo-F = 3.19, degree of freedom = 41, number of permutations = 999). Temperature and macronutrient concentrations appear as the main explanatory environmental variables of community composition (Fig. 2C), particularly in the large size fractions. The communities of the subtropical frontal region fall in between (Fig. 2B, Fig. S2) suggesting that a great share of the taxa derives from the mixing of the two regions. However, the proportion of species only found at the four sampled frontal stations (5.4%) is significantly higher than the average proportion of species only found in randomly chosen sets of four stations (and 3.2 ± 1.2% for 1000 random set of four stations). This implies that the frontal region harbors a particular ecological niche. The temperature and nitrate concentration distance matrix has a strong relationship with the ASVs Bray–Curtis dissimilarity matrix (Mantel statistic r = 0.829 and 0.780, respectively, *P* = 1x10^−4^) and doesn’t increase when including the other relevant environmental variables (ammonium, nitrite, DIP, Chl a, salinity, NPP, N_2_ fixation, particulate C and N concentrations and dissolved O_2_ concentration). This relationship is higher than the relationship with geographical location (i.e. the distance between stations, *r* = 0.594). In contrast, environmental variables select for species to a much lower extent in the small size fraction (Mantel statistic *r* = 0.320, 0.251, and 0.257 with all variables, nitrate only and temperature only, respectively), which appear to be almost as selective as geographical location (*r* = 0.200).

**Figure 3:**
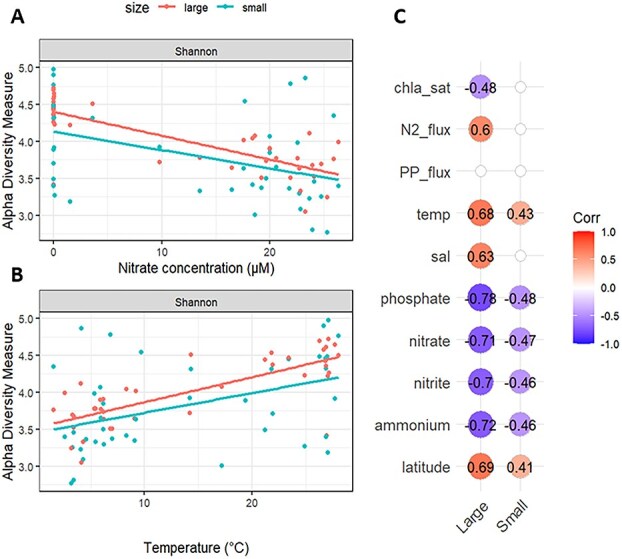
Alpha (local) diversity as a function of environmental parameters. Shannon diversity index for large and small size fraction as a function of nitrate concentration (A) and temperature (B). (C) Spearman correlation between Shannon diversity index and the main measured biogeochemical variables for large and small size fractions. Only regressions with *P*-value <0.005 are shown.

Local (alpha) diversity also correlated with environmental variables. In the large size fraction, both richness and Shannon indices showed significant negative correlations with all macronutrients measured (ammonium, nitrate, nitrite and DIP) and positives correlations with temperature, salinity and latitude (Spearman correlation, *P* < 0.05) (Fig. 3C). Similar patterns were observed for small size fractions but to a much lower extent with regression coefficient being about half lower as compared to large size fractions for parameters tested (Fig. 3C).

### N diffusion model

Application of the diffusion model to the cruise track shows that low DIN concentrations in the subtropical region set a cell size limit for N-based osmotrophic growth ranging between 2 and 18 μm for spherical cells at a growth rate of 0.2 d^−1^ (median 6 μm). Fast growers (>0.6 d^−1^) would have their cell size limited between 2 and 15 μm (median 5 μm). In the subantarctic region, limits are much higher than the largest osmotrophic cells known in the ocean, even at high growth rates (>2 d^−1^) implying that N diffusion is unlikely to play a role in limiting phytoplankton growth. Due to an excess in DIP relative to DIN (relative to a Redfield ratio of 16) noticeably in the subtropical region, none of the photosynthetic cells analysed appeared to be limited by P-based osmotrophic growth before being limited by N-based osmotrophic growth (data not shown).

**Figure 4:**
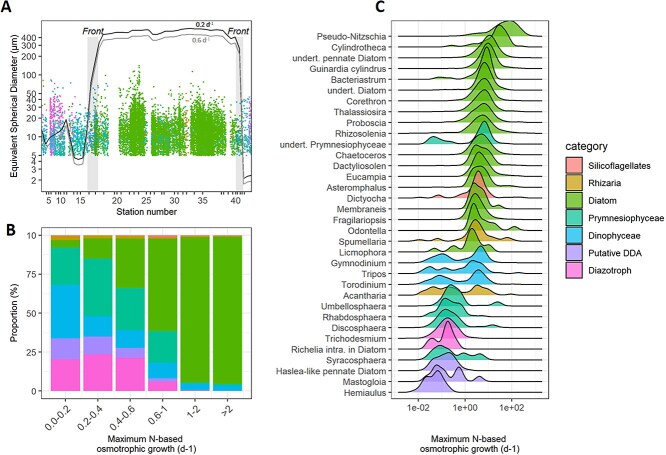
Maximum N-based osmotrophic growth rates according to the N diffusion model. (A) Equivalent spherical diameter (μm) of cells detected by automated imaging cell cytometer throughout the cruise duration. Station numbers are shown as reference. Only cells with taxonomic assignment to at least the class level were considered. Lines represent the modeled diameter (μm) of spherical cells for maximum N-based osmotrophic growth rates of 0.2 and 0.6 d^−1^. Note that lines (derived from spherical objects) and dots (derived from the imaged cells with diverse shapes) are not directly comparable. (B) Proportion of each taxonomic/functional group in ranges of maximum N-based osmotrophic growth. (C) Density plot of the maximum N-based osmotrophic growth rates for each taxa identified (with at least 10 cells). Colors code for all panels are shown in C. DDA: Diatom-Diazotroph association.

We confronted this model to the cells imaged during the cruise. We curated the 68 568 imaged objects acquired by the imaging flow cytometer and 15 738 were formally annotated as photosynthetic organisms to the class level at least. We applied the nutrient diffusion model to each of these organisms accounting local nutrient concentrations and their biovolumes and calculated their maximum N-based osmotrophic growth rates. The model predicts that the photosynthetic organisms imaged in the subantarctic region could maintain relatively high maximum N-based osmotrophic growth rates (>2 d^−1^) compared to the physiological growth limits of marine phytoplankton suggesting that N nutrient diffusion to the cells is unlikely to represent a limiting factor for growth (Fig. 4A). In contrast, 65% (n = 993) of the photosynthetic cells imaged in the subtropical region cells couldn’t meet a N-based osmotrophic growth rate above 0.2 d^−1^. Interestingly, 55% (*n* = 542) of the cells falling in this category are capable to circumvent N diffusion limitation by either diazotrophy (*Trichodesmium*, *Richelia intracellularis* associated with Diatoms, *n* = 202) or mixotrophy (*Dinophyceae, n* = 340) (Fig. 4B and C, Fig. S3A and B). Within the 451 remaining cells, 37 belong to the Diatom *Hemiaulus* genus and 10 to the *Chaetocerotaceae* family which has been shown to harbor the diazotrophic cyanobacteria *R. intracellularis* and *Calothrix*, respectively. We also observed 91 pennate diatoms with a N-based maximal osmotrophic growth rate below 0.2 d^−1^. Among these, a large proportion display morphological features of the genus *Haslea* which has recently been reported to host a diazotrophic endosymbiont belonging to the *Rhizobiales* order [50] (Fig. S4). The 18S dataset supports this taxonomic identification with *Haslea* ASVs found only in the N-depleted subtropical region and showing similar geographical pattern (Fig. S5A). Interestingly, cells belonging to the genus *Mastogloia* (n = 9) were also found to overcome N-based maximal osmotrophic growth rate threshold of 0.4 d^−1^ and showed similar dynamics to *Haslea*. This genus had never been reported to be directly associated with diazotrophs, but we observed one occurrence of association of *Mastogloia* and *R. intracellularis* in our imaging dataset (Fig. S4). When considering *Hemiaulus*, *Chaetoceros*, *Haslea* like cells and *Mastogloia* as DDAs, the proportion of diazotrophs and mixotrophic dinoflagellates in the categories of cell with a maximum N-based osmotrophic growth rates <0.2 d^−1^ increase from 55% to 68%, leaving only few cells annotated as pure osmotrophs. Interestingly, many of them (n = 238) were found to belong to Prymnesiophyceae, in particular *Rhabdosphaera, Umbellosphaera* and *Discosphaera*.

## Discussion

The findings presented here contribute significantly to our understanding of phytoplankton community dynamics in the southern Indian Ocean across a wide range of temperature and macronutrient concentrations. By employing a combination of amplicon sequencing, automated imaging flow cytometry and diffusive molecular fluxes modeling, this study provides new insights into how macronutrients exert a cell size-dependent role on structuring phytoplankton communities.

### Temperature and nutrient concentrations structure phytoplankton communities

The relatively high NPP in the subtropical region was likely sustained by high DIP and trace metal concentrations (iron concentration averaging 0.61 nM, Fig. S1A and B, Table S1) allowing diazotrophs to thrive and foster NPP [51]. This confirms previous reports which show the critical role of these nutrients in the formation of the so-called Madagascar blooms [52, 53]. As a result, NPP did not correlate with any of the main biogeochemical controlling parameters we measured, offering an opportunity to investigate the structure of phytoplanktonic communities of contrasted hydrological regimes with comparable productivity levels and avoiding possible biases arising from productivity-dependant patterns on community structure [54].

At the inter-basin scale, the results highlight the critical role of temperature and macronutrients as determinants of phytoplankton community composition and diversity. In the large size fraction, the dominance of diatoms in the colder, nutrient-rich subantarctic region contrasts with the prevalence of dinoflagellates in the warmer, nutrient-depleted subtropical region (Fig. 2). This pattern aligns with Margalef’s conceptual framework, which suggests that diatoms thrive in turbulent, nutrient-rich environments, while dinoflagellates and diazotrophs dominate stratified oligotrophic waters and is in line with Ocean wide observations [55]. The strong correlation between Bray–Curtis dissimilarity and environmental variables, particularly temperature and nitrate concentrations, underscores the selective role of these factors in shaping large (>3 μm) phytoplankton communities, in comparison to alternative drivers such as dispersal limitation or biotic interactions. Micronutrients, in particular iron or manganese, are also correlated with community structure suggesting a structuring role (see supplementary section for further discussion).

The subtropical frontal zone, with its intermediate temperature and nutrient conditions, emerges as a unique ecological niche in this study. The distinct phytoplankton communities observed in this region suggest that it serves as a transitional zone, where taxa from both subtropical and subantarctic regions coexist alongside frontal-specific species. The occurrence of frontal-specific taxa underscores the importance of environmental heterogeneity in promoting biodiversity. Comparable patterns have been described, suggesting that subtropical convergence zones act as hotspots for biodiversity, driven by the confluence of contrasting water masses and nutrient regimes [56–58].

### Cell size-dependent responses to nutrients fluxes

One of the most striking findings is the cell-size dependency of phytoplankton responses to environmental gradients. The weaker correlation in the small size fraction suggests that smaller phytoplankton are less influenced by environmental selection and may instead be shaped by biotic interactions (such as grazing and viral infections), dispersal dynamics [59], or ecological drift [34]. This finding aligns with the idea that smaller organisms, due to their higher surface-area-to-volume ratios, are more efficient at nutrient uptake and thus less constrained by macronutrient concentration [16]. Using a diffusion model we estimate the maximum osmotrophic growth rates of the cells imaged during the cruise. There are some caveats associated with this approach which are worth mentioning here. First, image segmentation is key to provide representative esd estimates. Intricate shape of some relatively large taxa (*Chaetoceros*, *Bacteriastrum*) strongly complicates this step, in particular as they tend not to be entirely on focus. Similarly, cells like *Umbellosphaera* are noticeably difficult to segment with intricate coccoliths shapes overlapping with each other. When possible we corrected manually the automatic segmentation steps for these species (see Material and method for details). Second, not only the SA/V ratio but also the shapes defines the diffusive fluxes of nutrients cells have access to [60]. Here, the maximum nutrients diffusion fluxes to the cell surface was modeled using mass transfer theory assuming cells as spheroids (Eq. 1) which is obviously not the case for many taxa. Alternative analytical solutions including cylindrical or rod shapes factors are available in the literature [61]. As an example, application of the model using cylindrical shape factor instead of spheroid shape with a length to diameter ratio of 10 (typical of *Trichodesmium*) increases the maximum osmotrophic growth rate by 15% [48, 60]. However, (i) since analytical solutions for all cellular shapes encountered in our study are not available, unless being individually modeled which would require tedious computing resources and (ii) changes of 15% wouldn’t modify the conclusions of our study, we decide to model diffusive fluxes assuming spheroid shapes only. Nevertheless, this emphasizes the potential of shapes deviating from pure spheres as a strategy for large cells to increase their osmotrophic access to nutrients [62]. Third, we did not measure urea and didn’t take it into account in our model. Urea can represent up to 50% of the N acquisition budget for phytoplanktonic communities, in particular for N-depleted regions of the Ocean [63–65]. Overall, urea would tend to allow larger cells to meet their N requirements by osmotrophy only. Assuming a diffusion coefficient for urea of 1.36x10^5^ cm^2^.s^−1^ [66] and a conservative concentration of 200 nmol L^−1^ in the subtropical region [63, 64] this would increase the cell size limit for osmotrophic growth from 2–15 to 6–20 μm for a growth rate of 0.6 d^−1^ in the subtropical region. Fourth, the current model does not account for the diverse nutrient acquisition strategies among phytoplankton, particularly for nitrogen, which can be taken up in various organic (e.g. urea, amino acids) and inorganic forms (e.g. NH₄^+^, NO₂^−^, and NO₃^−^). These uptake preferences have been shown to vary significantly across different cell size classes [33, 65, 67, 68].

Despite model caveats, preliminary results suggest that in the subantarctic region, where macronutrient concentrations are high, large phytoplankton can thrive without macronutrient diffusion constraints. In contrast, in the nutrient-depleted subtropical region, cell size appears to impose a significant constraint on nitrogen-based growth rates, with many large cells potentially circumventing this limitation through diazotrophy or mixotrophy. The presence of diazotrophic associations, such as those between diatoms and cyanobacteria (e.g. *Hemiaulus*-*Richelia* or putative *Haslea-Rhizobacter* associations) or between the Prymnesiophyceae *Braarudosphaera* and UCYN-A [51] (Fig. S5), highlights the convergent evolution mechanisms employed by phylogenetically distinct phytoplankton taxa to cope with N limitation [69, 70]. High DIP concentrations with depleted DIN created favorable conditions for diazotrophs in the subtropical region, supporting large phytoplankton cells and primary production.

These results emphasize that in the ocean, nutrient limitation plays a key role in structuring plankton communities. In nutrient-rich waters, fast-growing species like diatoms dominate, capitalizing on the abundant resources. However, in nutrient-depleted regions, competition intensifies, our results indicate that specialized taxa thrive by exploiting alternative strategies such as mixotrophy or diazotrophy [71]. This differentiation in resource use reduces competitive exclusion and allows a greater number of species to coexist. Moreover, nutrient limitation promotes microscale environmental heterogeneity [72]. These conditions would support a mix of generalists and specialists, further enhancing biodiversity. By fostering the coexistence of diverse taxa with contrasting nutrients acquisition strategies, nutrient limitation could play a crucial role in maintaining the resilience and functionality of phytoplankton communities in marine ecosystems. Such mechanisms were also highlighted by Boyd et al. [73], who linked nutrient gradients to enhanced ecological resilience and functional diversity in oceanic phytoplankton.

In this context temperature would act as a secondary driver of the phytoplankton community structure by selecting for best fitted taxa within cell size ranges adapted to local nutrient concentrations [14, 17, 74]. Other environmental parameters (e.g. NPP, trace metal concentrations) showed no or weaker correlations suggesting they play a secondary role at the inter-regional scale. However, when zooming in at the intra-regional scale, micronutrients often appear to play a critical role in shaping community structure and stimulating primary production both in the subtropical and in the subantarctic regions sampled [52, 75, 76].

### The case of Prymnesiophyceae

Finally, the ecological and biogeochemical roles of less-studied taxa, such as the Prymnesiophyceae observed in this study, remain poorly understood. However, under N depleted conditions, many Prymnesiophyceae, such as *Chrysochromulina* and *Prymnesium* species, exhibit phagotrophic feeding behaviors. These taxa actively graze on bacteria or assimilate dissolved organic matter, as demonstrated in experimental studies [77, 78]. *Prymnesium parvum* thrives under phosphorus-limited conditions by supplementing its nutrient requirements through heterotrophy, enabling it to maintain cellular functions and outcompete strictly autotrophic taxa [79]. *Chrysochromulina* was identified in our 18S dataset, at significantly higher abundances on average in the N depleted subtropical region than in the subantarctic region (Fig. S5B). The Prymnesiophyceae *Braarudosphaera* fixes N_2_ by hosting a potential organelle -so called nitroplast- from a cyanobacterial origin [80] and also shows mixotrophic behavior [81]. *Braarudosphaera* was identified in our 18S data noticeably in the subtropical region at relatively high abundances (Fig. S5C) which echoes on data reported by Chowdhury et al. [51] who found similar patterns for UCYN-A1 using nifH based amplicon sequencing and qPCR. However these examples remain relatively limited when confronted with the wide diversity of Prymnesiophyceae species and questions the extent of alternative nutrition strategies (e.g. mixotrophy/diazotrophy) in this class. Continuing efforts in cultivation-based studies and advanced omics approaches, such as metagenomics and transcriptomics, will improve our understanding of their physiology and ecological and biogeochemical roles in the ocean.

### Future directions and concluding remarks

While this study provides valuable insights, it also highlights several challenges and avenues for future research. One key challenge is disentangling the relative contributions of environmental selection and biotic interactions to phytoplankton community dynamics. As ocean stratification intensifies, nutrient-limited regions are expected to expand, making it essential to understand how these conditions affect phytoplankton communities. Our results emphasize on the critical role of macronutrient concentrations (in particular nitrogen) in explaining the structure of the communities and their size distribution north of the subtropical front of the southern sector of the Indian Ocean, arguing for the presence of resource-based competitive exclusion in plankton communities [35]. The weaker correlation between environmental variables and community composition in the small size fraction suggests that biotic factors such as grazing and viral infections may play a larger role than previously recognized. Understanding these interactions will require novel experimental and modeling approaches that integrate biotic and abiotic factors.

Another important avenue for future research is the role of micronutrients, such as iron, manganese and nickel, in shaping phytoplankton communities. While concentration based micronutrients data provide little evidence on their direct use plankton communities, their importance for processes such as diazotrophy and mixotrophy warrants further investigation in this region [52, 82] and in other regions known to harbor blooms which are largely driven by micronutrient concentrations.

Using the southern Indian Ocean as a testbed, this study underscores the complex interplay between temperature, nutrients, and phytoplankton community dynamics. The findings highlight the importance of cell size as a mediator of ecological responses and reveal distinct community structures and adaptive strategies in response to environmental gradients. As climate change continues to alter ocean conditions, understanding these dynamics will be crucial for predicting the future of marine ecosystems, the biogeochemical cycling of elements and climate.

## Supplementary Material

Supplementary_text_and_figures_ycaf195

Table_S1_metadata_ycaf195

Table_S2_eco_taxa_annotation_category_ycaf195

## Data Availability

All biogeochemical data generated or analysed during this study are included in Table S1. Imaging data are available on Ecotaxa platform (https://ecotaxa.obs-vlfr.fr/prj/13402) and taxonomic categories and their associated trophic strategies are available in Table S2. Raw sequencing data are deposited on the European Nucleotide Archive (ENA, https://www.ebi.ac.uk/ena) with the accession number PRJEB89894.
