# Supplementary material for: Nitrogen concentration shapes the size structure and the functional diversity of phytoplankton communities in the southern Indian Ocean
Source: ISME Commun. 2025 Nov 20;5(1):ycaf195. doi: 10.1093/ismeco/ycaf195 (PMC12663791; doi:10.1093/ismeco/ycaf195)
Supplement: Supplementary_text_and_figures_ycaf195 [file supplementary_text_and_figures_ycaf195.pdf]

Supplementary Information

Nitrogen concentration shapes the size structure and the functional diversity of phytoplankton communities in the southern Indian Ocean

Hugo Berthelot[1,2,3,*], Joanna Zukowska[2], Nicolas Henry[4,5], Cyril Noël[6], Melilotus Thyssen[7], Karine Leblanc[7], Hélène Planquette[1], Jean-François Maguer[1], Rainer Pepperkok[2], Colomban de Vargas[4,5], Nicolas Cassar[1,8]

[1]CNRS, Université de Brest, IRD, Ifremer, LEMAR, F-29280 Plouzané, France

[2]Cell Biology and Biophysics Unit, European Molecular Biology Laboratory, 69117 Heidelberg, Germany

[3]Now at IFREMER, DYNECO, Pelagos Laboratory, F-29280 Plouzané, France

[4]CNRS, Sorbonne Université, FR2424, ABiMS, Station Biologique de Roscoff, Roscoff, 29680 France

[5]Research Federation for the Study of Global Ocean Systems Ecology and Evolution, FR2022/GOSEE, 3 rue Michel-Ange, Paris, 75016 France

[6]Ifremer, IRSI, SeBiMER Service de Bioinformatique de l'Ifremer, F-29280 Plouzané, France

[7]Aix Marseille University, Université de Toulon, CNRS, IRD, MIO UM 110, 13288, Marseille, France.

[8]Division of Earth and Climate Sciences, Nicholas School of the Environment, Duke University, Durham, NC, USA

This file includes:

-Extended Methods

-Supplementary Figures S1-S6

Extended Methods

N_2 fixation and net primary production

Samples for measuring net primary production (NPP) and N_2 fixation rates were collected from the underway sampling system into sets of four 2.3-liter acid-washed polycarbonate bottles. Three of these bottles were spiked with 2.3 mL of a ^{13}C-labeled bicarbonate solution ($NaH^{13}CO_2$; >98%, Sigma Aldrich, 10 atom% final ^{13}C abundance) and 2.3 ml of $^{15}N_2$ (^{15}N isotopic abundance of 99.7%, Eurisotop, Saclay, France) using the bubble method to maximize $^{15}N_2$ isotopic enrichment and improve detection limits. The bottles were inverted at least 60 times (for at least 2 minutes) before incubation to ensure rapid isotopic equilibrium. The fourth bottle was left unamended and used as a control. All bottles were incubated for 24 hours in an on-deck incubator with circulating surface water and a blue filter to simulate light conditions at the sampling depth. The average temperature change between the start and end of the incubation was 1.2°C. Following incubation, 12 ml of water were siphoned from the bottles into Exetainer tubes and preserved with $HgCl_2$ for $^{15}N_2$ isotopic abundance analysis. The remaining contents of the bottles were filtered onto combusted (450°C, 4 h) 25-mm diameter glass fiber filters (Whatman, London, UK). The filters were stored at −20°C before being dried for downstream analysis (24 h, 60°C). The $^{15}N_2$ isotopic abundance in water was measured within 6 months using a membrane inlet mass spectrometer as described by Kana et al. [1]. The particulate carbon and nitrogen isotopic enrichment (^{13}C and ^{15}N) was measured using an elemental analyzer (Flash EA C/N, Thermofisher Scientific) coupled with an isotope ratio mass spectrometer (IRMS Delta plus, Thermofisher Scientific). N_2 fixation rates and NPP were calculated according to Montoya et al. [2].

18s rRNA amplicon sequencing

Samples for metabarcoding of the 18S rRNA gene were collected at each station in 4.5-liter acid-washed polycarbonate bottles after prefiltration at 200 µm to exclude large metazoans, then filtered sequentially through a 3.0 µm polycarbonate filter and a 0.2 µm Sterivex cartridge, generating two size fractions (0.2–3.0 µm and 3.0–200 µm, referred to as small and large size fractions, respectively). Size fractions were chosen to match with other Ocean wide projects [3, 4]. Both the filter and cartridge were immediately stored at -80°C. DNA was extracted using the Nucleospin Plant II Mini Kit following the manufacturer's standard protocol. The V4 region of the 18S rRNA gene was amplified using staggered primers sets based on TAReuk454FWD1 and

TAReukREV3 [5]. The PCR mixes (10µl) contained 0.02 u/µl of Master Mix Phusion High-Fidelity DNAPolymerase (Finnzymes; ThermoFisher), 5 µM of each primer, 3% dimethylsulphoxide, 200 µM dNTP and 1 ng of sample DNA. The PCR program consisted in initial denaturation step at 98°C for 30s, 10 cycles of denaturation at 98°C for 10 s, annealing at 53°C for 30s and elongation at 72°C for 30s, then 20 similar cycles but with 62°C annealing temperature, and a final step at 72°C for 7 min. PCR blanks were used to identify contaminations in the samples. Amplification results were checked by gel electrophoresis. Pooled staggered samples were sequenced in paired-end mode (2 × 250bp) on a MiSeq (Illumina, San Diego, CA, USA) at EMBL GeneCore facility. Raw data were processed using the SAMBA (Standardized and Automated MetaBarcoding Analyses) open-source workflow (https://gitlab.ifremer.fr/bioinfo/workflows/samba). In summary, the workflow consists of filtering, trimming and merging reads using QIIME 2 and DADA2 [6, 7], clustering ASV sequences with dbOTU3 [8] and removing contamination with the R package microDecon [9]. The ASV assignments were performed using a naive Bayesian approach (`feature-classifier classify-sklearn` plugin from QIIME 2) with the PR2 database as a reference (version 5.1.0). Out of the 3.8 x 10^6 reads belonging to 4705 ASVs, from 82 samples, we selected photosynthetic taxa (including Chlorophyta, Cryptophyta, Haptophyta and Gyrista) and Dinophyceae and ASVs with fewer than 2 reads were removed to limit sequencing artifacts, leaving 1.7 x 10^6 reads belonging to 1640 ASVs. The functional group "Diatoms" includes the classes Bacillariophyceae, Coscinodiscophyceae, Diatomeae_X and Mediophyceae. Rarefaction curves were checked which all showed near saturation (Fig. S6A). We fitted our data to the truncated Preston log-normal model and the Preston veil indicated that our sampling strategy covered >99.7% of the modeled total richness (Fig. S6B). Raw data were deposited on the European Nucleotide Archive (ENA, https://www.ebi.ac.uk/ena) with the accession number PRJEB89894.

Automated microscopy imaging

An automated imaging flow cytometer (CytoSense CytoBuoy) [10] was installed on the underway sampling system. It sampled seawater in a dedicated 200 mL chamber which isolates seawater every 2h. Between two consecutive samples the chamber was flushed continuously by the seawater circuit of the ship inorder to clean and renew the seawater. A sheath fluid made of 0.1 µm filtered seawater stretched the sample in order to separate, align, center and drive the individual particles through a laser beam (488 nm wavelength). The image acquisition targeting chlorophyll rich objects above an equivalent spherical diameter (esd) of ca 7 µm was previously

set using the Cytoclus software. Acquired images had a pixel size of 0.3 µm, and consisted in a random selection of particles passing in front of the laser beam within the predefined set, representing up to 90% of the counted particles within the >15 µm size class. Objects within each image were segmented and cell surface and biovolumes were estimated following Moberg and Sosik [11][64] guidelines using the "EBImage" and "EBImageExtra" R packages. In short, cell biovolumes were estimated using distance maps based on initial two-dimensional segmentation. Third dimension calibration was made against imaged spherical bubbles. Segmentation was found to overestimate systematically the volume of intricate shape small taxa (*Umbellosphaera*, *Discosphaera* and *Rhabdosphaera*). In this case manual corrections were applied. Cells with esd below 5 µm were withdrawn from the dataset. Morphological features were extracted using the "computeFeatures" function from "EBImage" R package. Images and associated morphological features were deposited on Ecotaxa (https://ecotaxa.obs-vlfr.fr/gui/prj/13402). Taxonomic assignment was performed individually for each image, with the help of the Ecotaxa built-in classifier. Trophic strategy was assigned to each taxa and is available as supplementary data (Table S2).

Extended Discussion

Micronutrients

Micronutrients, in particular iron or manganese, play a crucial role in regulating primary production in the southern sector of the Indian Ocean, both in the subtropical waters [12] and in the subantarctic waters [13]. Atmospheric deposition, hydrothermal inputs, and upwelling processes serve as key sources of iron, though its bioavailability is often constrained by rapid scavenging and complexation with organic ligands. Studies have shown that episodic iron enrichment events, such as those associated with natural upwelling or anthropogenic sources, can stimulate phytoplankton blooms [14], thereby enhancing carbon sequestration and influencing regional biogeochemical cycles. The relatively small number of stations where micronutrients were measured in our study (13 stations out of 43) hinders drawing clear conclusions on their role in community composition. However, significant correlations between manganese, nickel and community composition were observed, albeit in opposing directions (Fig. S1B). Nickel protects *Trichodesmium* urease (enzyme responsible for N_2 fixation process) from oxidative stress generated during photosynthesis [15]. Nickel is also required by urease enzyme, responsible for urea uptake, in particular for Dinophyceae [16]. The high abundances of *Trichodesmium sp.* and

Dinophyceae observed in subtropical waters may have led to high demand in nickel and could explain the low concentrations observed compared to subantarctic waters. Manganese follows an opposite pattern with higher concentrations in the subtropical water compared to subantarctic waters. Manganese has been postulated to play a similar role than Nickel in the protection of *Trichodesmium* nitrogenase, but this role has also been questioned based on culture and in-situ experiments [15, 17, 18]. Our data would rather argue for the later case with high concentrations reflecting a low demand. However, this points out the difficulty to interpret concentration data as the interplay between biogenic elements and plankton communities are dynamic and flux-based rather than static and stock-based, especially in micro- or macro-nutrient limited environments such as the regions studied here.

Reference

1. Kana TM et al. Membrane Inlet Mass Spectrometer for Rapid High-Precision Determination of N_2, O_2, and Ar in Environmental Water Samples. *Analytical Chemistry* 1994;**66**:4166–4170. https://doi.org/10.1021/ac00095a009
2. Montoya JP, Voss M, Kahler P, Capone DG. A Simple, High-Precision, High-Sensitivity Tracer Assay for N_2 Fixation. *Applied and environmental microbiology* 1996;**62**:986–93.
3. Gorsky G et al. Expanding Tara Oceans Protocols for Underway, Ecosystemic Sampling of the Ocean-Atmosphere Interface During Tara Pacific Expedition (2016–2018). *Front Mar Sci* 2019;**6**. https://doi.org/10.3389/fmars.2019.00750
4. Sánchez P et al. Marine picoplankton metagenomes and MAGs from eleven vertical profiles obtained by the Malaspina Expedition. *Sci Data* 2024;**11**:154. https://doi.org/10.1038/s41597-024-02974-1
5. Stoeck T et al. Multiple marker parallel tag environmental DNA sequencing reveals a highly complex eukaryotic community in marine anoxic water. *Molecular Ecology* 2010;**19**:21–31. https://doi.org/10.1111/j.1365-294X.2009.04480.x
6. Callahan BJ et al. DADA2: High-resolution sample inference from Illumina amplicon data. *Nat Methods* 2016;**13**:581–583. https://doi.org/10.1038/nmeth.3869
7. Bolyen E et al. Reproducible, interactive, scalable and extensible microbiome data science using QIIME 2. *Nat Biotechnol* 2019;**37**:852–857. https://doi.org/10.1038/s41587-019-0209-9
8. Olesen SW, Duvallet C, Alm EJ. dbOTU3: A new implementation of distribution-based OTU calling. *PLoS ONE* 2017;**12**:e0176335. https://doi.org/10.1371/journal.pone.0176335
9. McKnight DT et al. microDecon: A highly accurate read-subtraction tool for the post-sequencing removal of contamination in metabarcoding studies. *Environmental DNA* 2019;**1**:14–25. https://doi.org/10.1002/edn3.11
10. Dubelaar GBJ, Gerritzen PL. CytoBuoy: a step forward towards using flow cytometry in operational oceanography. *Sci Mar* 2000;**64**:255–265. https://doi.org/10.3989/scimar.2000.64n2255
11. Moberg EA, Sosik HM. Distance maps to estimate cell volume from two-dimensional plankton images. *Limnology & Ocean Methods* 2012;**10**:278–288. https://doi.org/10.4319/lom.2012.10.278
12. Chinni V, Singh SK, Bhushan R, Rengarajan R, Sarma VVSS. Spatial variability in dissolved iron concentrations in the marginal and open waters of the Indian Ocean. *Marine Chemistry* 2019;**208**:11–28. https://doi.org/10.1016/j.marchem.2018.11.007
13. Browning TJ, Achterberg EP, Engel A, Mawji E. Manganese co-limitation of phytoplankton growth and major nutrient drawdown in the Southern Ocean. *Nat Commun* 2021;**12**:884. https://doi.org/10.1038/s41467-021-21122-6
14. Weis J et al. Southern Ocean Phytoplankton Stimulated by Wildfire Emissions and Sustained by Iron Recycling. *Geophysical Research Letters* 2022;**49**:e2021GL097538. https://doi.org/10.1029/2021GL097538
15. Chen C et al. Nickel superoxide dismutase protects nitrogen fixation in *Trichodesmium*. *Limnol Oceanogr Letters* 2022;**7**:363–371. https://doi.org/10.1002/lol2.10263

16. Dyhrman ST, Anderson DM. Urease activity in cultures and field populations of the toxic dinoflagellate *Alexandrium*. 2003. 2003.
17. Held NA et al. Mechanisms and heterogeneity of in situ mineral processing by the marine nitrogen fixer *Trichodesmium* revealed by single-colony metaproteomics. *ISME Communications* 2021;**1**:35. https://doi.org/10.1038/s43705-021-00034-y
18. Ho T-Y. Nickel limitation of nitrogen fixation in *Trichodesmium*. *Limnology & Oceanography* 2013;**58**:112–120. https://doi.org/10.4319/lo.2013.58.1.0112

Supplementary figures

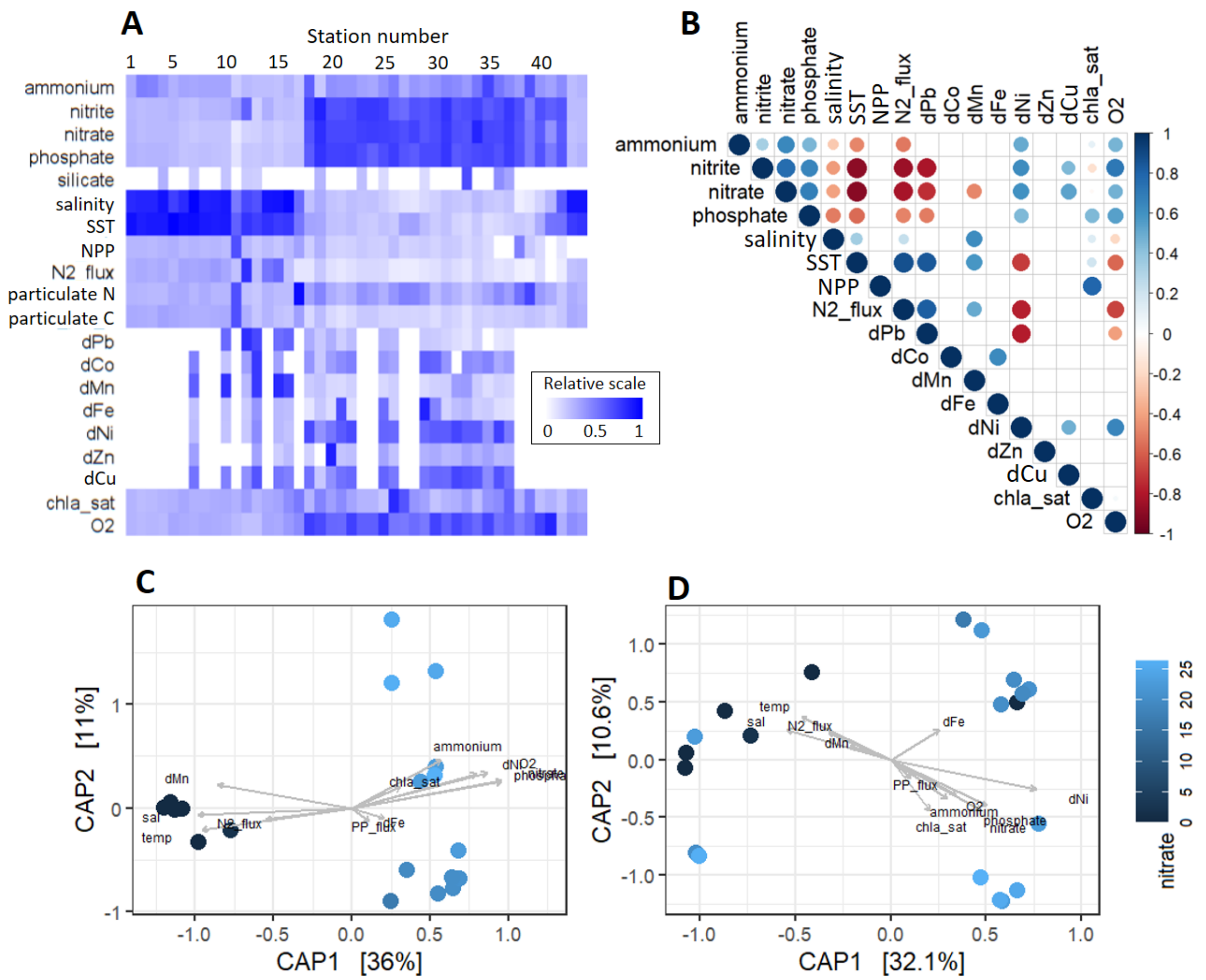

Figure S1. (A) Normalized heat map of the different biogeochemical parameters measured as a function of the stations. NPP: Net Primary Production. dPb, DCo, dFe, DNi, dZn, dCu : dissolved micronutrients concentrations. Chl a: chlorophyll a concentration. O_2: dissolved O_2 concentration (B) *Heatmap of the correlations between environmental variables including dissolved concentration of the micronutrient (Spearman correlation, only significant correlation at the threshold of p<0.005 are shown). Analysis excluded stations if only one parameter is missing*. Silicates are excluded to avoid excluding too many stations from the analysis. *Constrained Analysis of Principal coordinates (CAP) based on the environmental parameters (including micronutrients), for large (C) and small (D) size fractions. Raw data are available as supplementary data file (Table S1).*

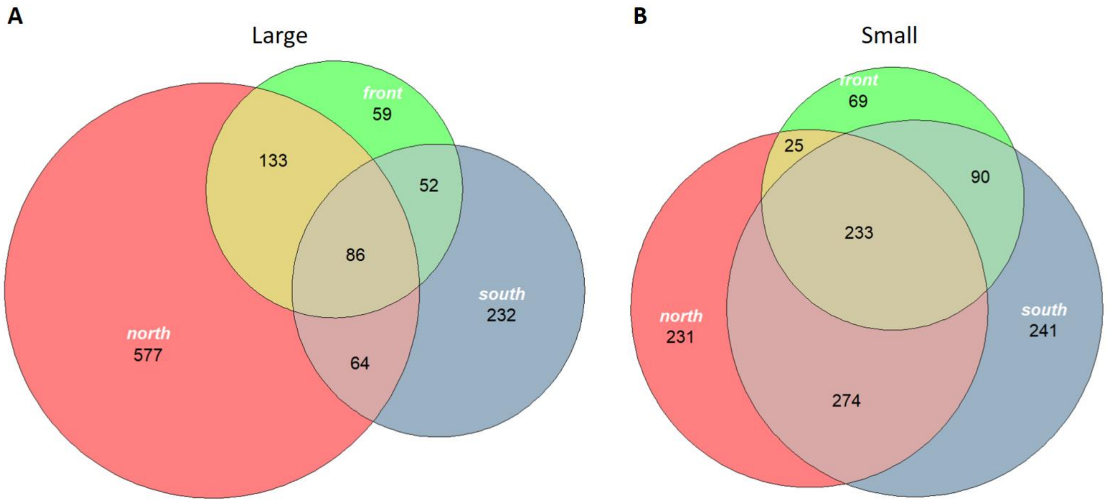

Figure S2. Venn diagrams of the Amplicon Sequence Variant (ASV) detected in the large (A) et small (B) size fractions in each to the regions identified.

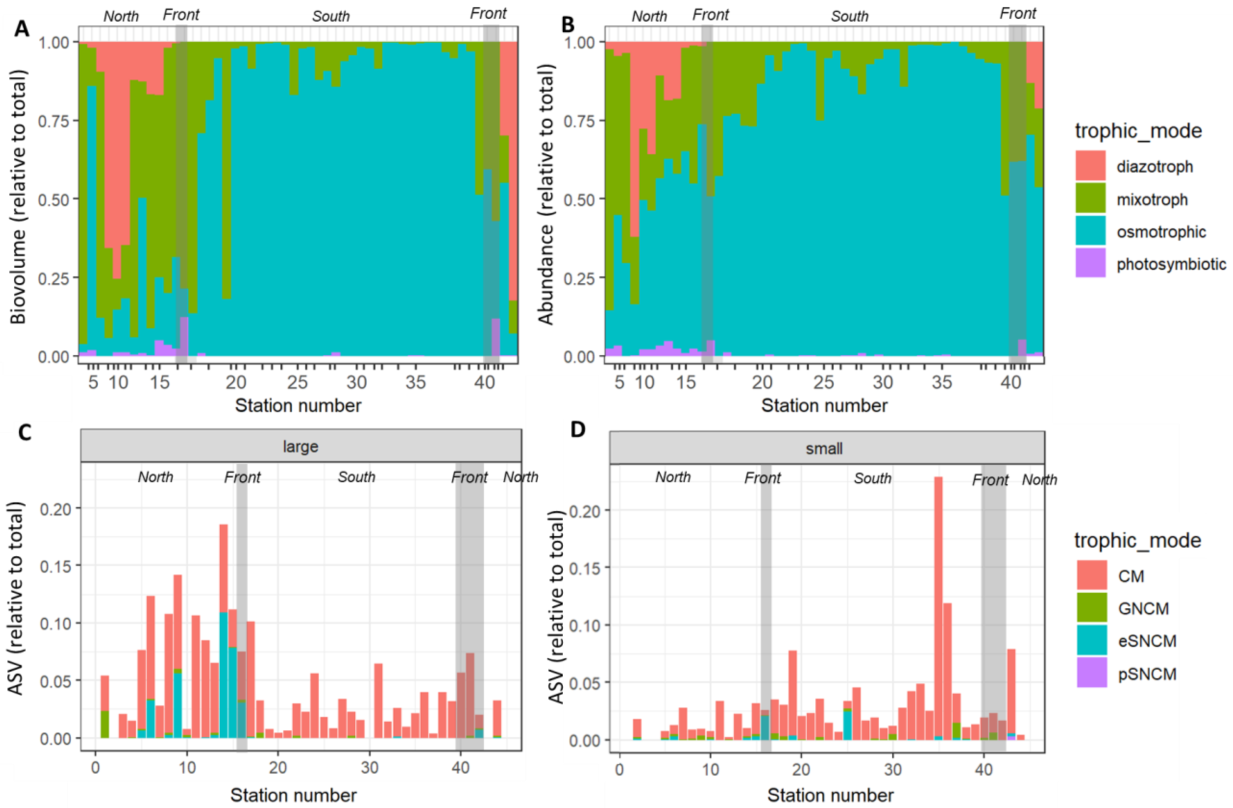

Figure S3: Relative biovolumes (A) and abundances (B) of particles imaged by imaging cytometer along the cruise as a function of the sampling date. The data were binned on a 24h-basis. Only particles with taxonomy assigned to photosynthetic organism are shown. Relative abundances of the different mixoplankton groups analyzed by amplicon sequencing of the V4 region of the 18S gene in the large (C) and small size fractions (D) according to the PR2. CM: constitutive mixotrophy, GNCM: Generalist Non-Constitutive Mixoplankton, eSNCM: endosymbiotic Specialist Non-Constitutive Mixoplankton, pSNCM: plastidic Specialist Non-Constitutive Mixoplankton.

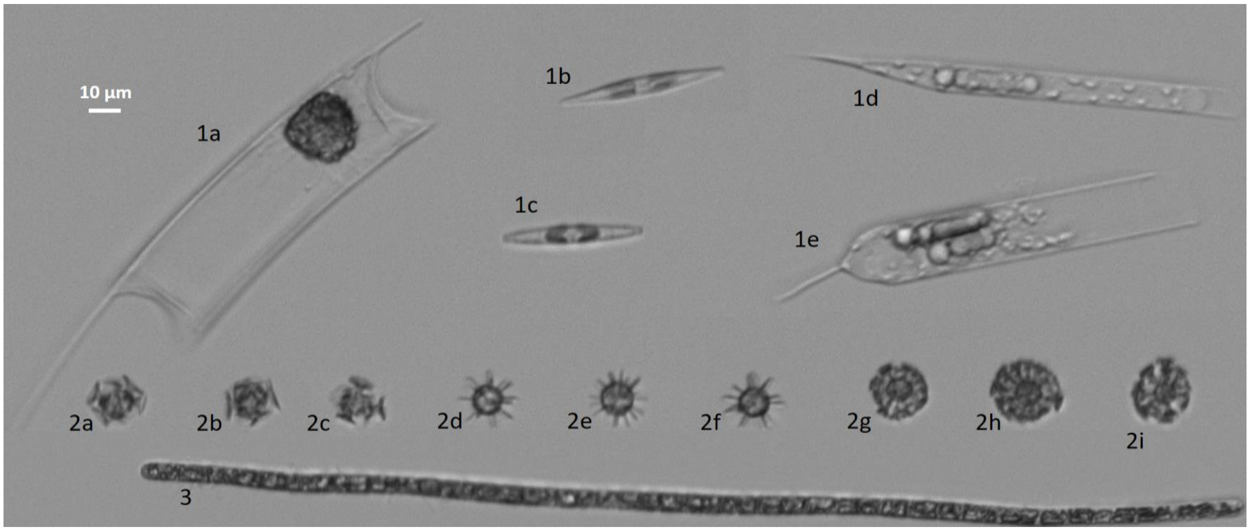

Figure S4. Microscopic photographs of phytoplankton imaged by the cytosense imaging cytometer. 1a, *Hemiaulus sp.* 1b and 1c, *Haslea*-like cells. 1d and 1e, *Richelia intracellularis* in association with *Rhizosolenia sp.* and *Guinardia sp.*, respectively. 2 Prymnesiophyceae and the genus *Umbellosphaera* (2a-c), *Rhabdosphaera* (2d-e) and *Discophaera*(2g-i). 3. *Trichodesmium sp.* filament.

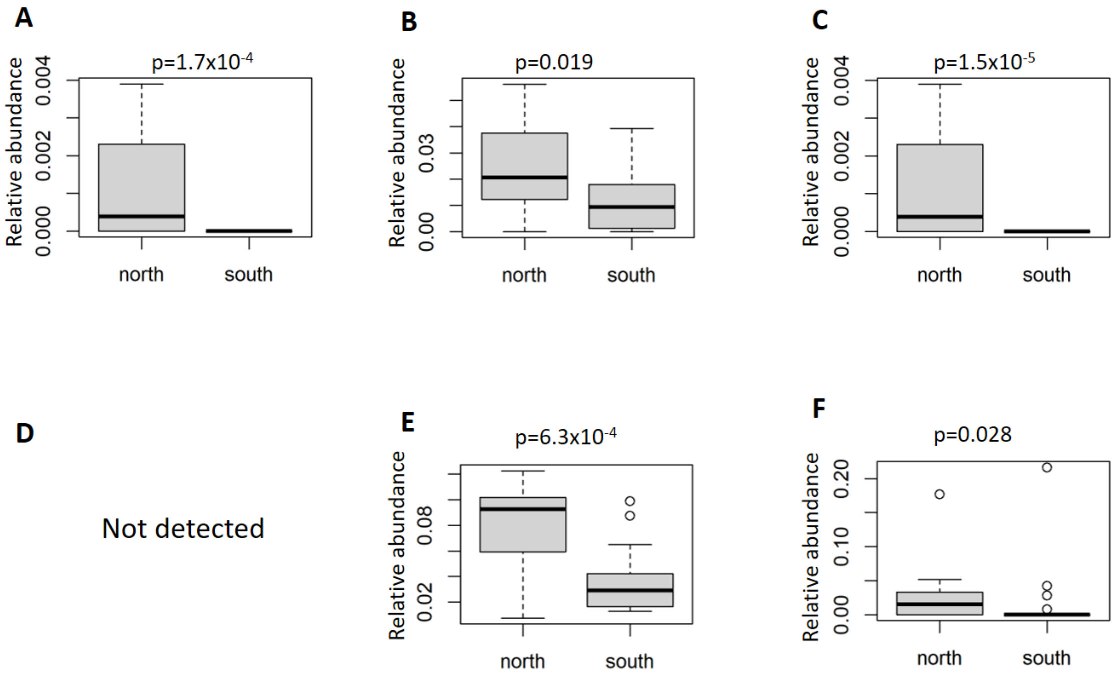

Figure S5. Boxplot of the relative abundances of photosynthetic amplicon sequence variant (ASVs) assigned to *Haslea* (A,D), *Chrysochromulina* (B,E) and *Braarudosphaera* (C,F) for the large (A,B and C) and the small (D, E and F) size fractions. No *Haslea* were detected in the small size fraction. Significant differences between regions were tested using Wilcoxon test and associated p values are shown.

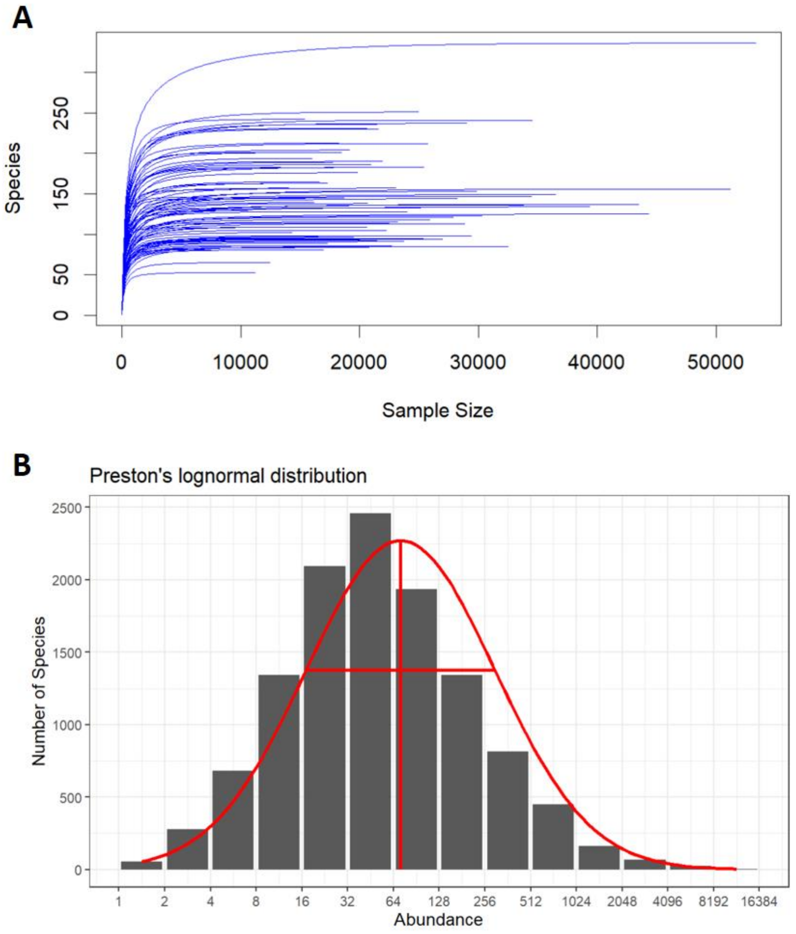

Figure S6. (A) Rarefaction curve for the Amplicon Sequence Variants (ASVs) sampled at each station. (B) ASVs abundance distribution and fit to the Preston log-normal model using maximized likelihood to log2 abundances.
